# Isothiocyanate-enriched moringa seed extract alleviates ulcerative colitis symptoms in mice

**DOI:** 10.1371/journal.pone.0184709

**Published:** 2017-09-18

**Authors:** Youjin Kim, Alex G. Wu, Asha Jaja-Chimedza, Brittany L. Graf, Carrie Waterman, Michael P. Verzi, Ilya Raskin

**Affiliations:** 1 Nutrasorb, LLC., Freehold, New Jersey, United States of America; 2 Department of Plant Biology, Rutgers, The State University of New Jersey, New Brunswick, New Jersey, United States of America; 3 Department of Genetics and the Human Genetics Institute of New Jersey, Rutgers, The State University of New Jersey, Piscataway Township, New Jersey, United States of America; 4 Department of Nutrition, University of California-Davis, Davis, California, United States of America; Institute of Biochemistry and Biotechnology, TAIWAN

## Abstract

Moringa (*Moringa oleifera* Lam.) seed extract (MSE) has anti-inflammatory and antioxidant activities. We investigated the effects of MSE enriched in moringa isothiocyanate-1 (MIC-1), its putative bioactive, on ulcerative colitis (UC) and its anti-inflammatory/antioxidant mechanism likely mediated through Nrf2-signaling pathway. Dextran sulfate sodium (DSS)-induced acute (n = 8/group; 3% DSS for 5 d) and chronic (n = 6/group; cyclic rotations of 2.5% DSS/water for 30 d) UC was induced in mice that were assigned to 4 experimental groups: healthy control (water/vehicle), disease control (DSS/vehicle), MSE treatment (DSS/MSE), or 5-aminosalicyic acid (5-ASA) treatment (positive control; DSS/5-ASA). Following UC induction, water (vehicle), 150 mg/kg MSE, or 50 mg/kg 5-ASA were orally administered for 1 or 2 wks. Disease activity index (DAI), spleen/colon sizes, and colonic histopathology were measured. From colon and/or fecal samples, pro-inflammatory biomarkers, tight-junction proteins, and Nrf2-mediated enzymes were analyzed at protein and/or gene expression levels. Compared to disease control, MSE decreased DAI scores, and showed an increase in colon lengths and decrease in colon weight/length ratios in both UC models. MSE also reduced colonic inflammation/damage and histopathological scores (modestly) in acute UC. MSE decreased colonic secretions of pro-inflammatory keratinocyte-derived cytokine (KC), tumor necrosis factor (TNF)-α, nitric oxide (NO), and myeloperoxidase (MPO) in acute and chronic UC; reduced fecal lipocalin-2 in acute UC; downregulated gene expression of pro-inflammatory interleukin (IL)-1, IL-6, TNF-α, and inducible nitric oxide synthase (iNOS) in acute UC; upregulated expression of claudin-1 and ZO-1 in acute and chronic UC; and upregulated GSTP1, an Nrf2-mediated phase II detoxifying enzyme, in chronic UC. MSE was effective in mitigating UC symptoms and reducing UC-induced colonic pathologies, likely by suppressing pro-inflammatory biomarkers and increasing tight-junction proteins. This effect is consistent with Nrf2-mediated anti-inflammatory/antioxidant signaling pathway documented for other isothiocyanates similar to MIC-1. Therefore, MSE, enriched with MIC-1, may be useful in prevention and treatment of UC.

## Introduction

Ulcerative colitis (UC), a type of inflammatory bowel disease (IBD), is a chronic intestinal disorder characterized by recurrent inflammation of the gastrointestinal tract. It affects over 1.6 million people in the United States [1–3]. The etiopathogenesis of IBD involves a complex interplay of genetic, environmental, microbial, and immune factors, which result in altered barrier properties of the mucous and epithelial layers. These alterations can allow luminal toxins, pathogens, and antigens to penetrate the intestinal mucosa and elicit an overproduction of pro-inflammatory cytokines and trafficking of effector leukocytes into the intestinal mucosa. This can ultimately lead to uncontrolled and exaggerated intestinal inflammation [4].

Pharmacological intervention is one of the key approaches for UC treatment. Anti-inflammatory drugs such as corticosteroids, 5-aminosalicyic acid (5-ASA) derivatives, and immune-suppressants can reduce mucosal inflammation and UC-related symptoms [5]. While these therapies are often effective, their long-term use is limited by significant side effects including immune suppression [6]. Hence, there is a need to discover new therapeutic options for UC that are safe and capable of sustaining clinical remission, while improving gut mucosal healing. In this respect, dietary and medicinal plants containing bioactive phytochemicals are emerging as promising alternatives to drug therapies for prevention and treatment of UC [7].

Moringa (*Moringa oleifera* Lam.) is a fast growing, drought-tolerant tree that thrives in tropical and subtropical regions. It belongs to the Moringaceae family within the order Brassicales which includes broccoli and other cruciferous vegetables of the Brassicaceae family. Traditionally, moringa is renowned for its nutritional value and medicinal benefits. All parts of the plant (i.e., leaves, seeds, roots, bark, flowers) are edible and used for treatment of a number of acute and chronic ailments such as inflammation, infection, hypertension, and diabetes [8]. The known toxicity of moringa is mostly limited to a moringa root, which contains alkaloid spirochin, a potential neuro-paralytic toxin that can cause paralysis and death [8]. Recently, medicinal benefits of moringa have been confirmed in multiple *in vitro* and *in vivo* studies [9–11]. Observed pharmacological effects of moringa, linked to its anti-inflammatory, anti-bacterial, and antioxidant properties, are attributed to the presence of phytoactive compounds such as flavonoids, phenolic acids, and, most notably, its unique glucosinolates [12]. Moringa glucosinolates can be converted, via myrosinase, to highly bioactive and stable moringa isothiocyanates (MICs) [9, 12], of which MIC-1 (4-[(α-L-rhamnosyloxy)benzyl] isothiocyanate) is the most abundant MIC in moringa seeds. Isothiocyanates from Brassicaceae plants, taxonomically related to moringa, are well-known anti-inflammatory compounds that likely act via activation of nuclear factor erythroid 2-related factor 2 (Nrf2), a key transcription factor involved in anti-inflammatory and antioxidant responses [13, 14]. A growing body of evidence has supported the anti-inflammatory effect of MIC-1 by suggesting that MIC-1 inhibited nuclear factor-kappa B (NF-κB) expression in nude mice [15], and attenuated gene expression of inducible nitric oxide synthase (iNOS) and interleukin-1β (IL-1β), as well as production of nitric oxide (NO) and tumor necrosis factor-α/β (TNF-α/β) in RAW macrophages [12, 16]. An MIC-1-enriched moringa leaf extract also reduced IL-1β and TNF-α levels in plasma and various tissues, and ameliorated weight gain, insulin resistance, and hepatic gluconeogenesis in obese mice [17]. Furthermore, MIC-1 is known to activate NAD(P)H quinone oxidoreductase 1 (NQO1) [18], one of the phase II detoxifying enzymes that are directly regulated by Nrf2 [13]. Nrf2-deficient mice are susceptible to UC and colorectal cancer, which was correlated with downregulation of antioxidant/phase II detoxifying enzymes and upregulation of pro-inflammatory cytokines and related biomarkers [19]. Therefore, Nrf2 signaling has been suggested to play a role in mediating the inflammatory response involved in UC.

To examine the *in vivo* efficacy of potential therapeutic agents, various colitogenic substances, such as dextran sulfate sodium (DSS), di-/tri-nitrobenzene sulfonic acid, oxazolone, or acetic acid, have been used to establish chemically-induced UC models [20]. Of the experimental UC models, DSS-induced UC in mice is most commonly used due to its simplicity, affordability, high degree of uniformity, and reproducibility [21]. The DSS model generally mimics the common clinical features of inflammation and histopathology observed in human UC [22]. In this experimental model, changes in concentrations and durations of DSS, delivered in drinking water, can mimic acute or chronic UC [23].

Earlier, our laboratory developed a moringa leaf extract enriched in several MICs, averaging 3–5% (*w/w*), by extracting fresh moringa leaves in water and utilizing endogenous myrosinase to convert the precursor glucosinolate to MICs, and reported biological activities of this extract and stability of MICs contained [12, 17–18]. Subsequently, we developed a method for producing 40–50% (*w/w*) MIC-1- enriched moringa seed extract (MSE) and biochemically characterized this extract [24]. We also completed a 14-day repeated-dose oral toxicological evaluation of MSE in male/female rats, in which the no observation of adverse effect level (NOAEL) of MSE was estimated to equal 100 mg/kg body weight (bw)/d MIC-1. This companion paper provides evidence that MIC-1 is the main anti-inflammatory bioactive in MSE responsible for reductions of carrageenan-induced edema *in vivo* and inflammatory responses in lipopolysaccharide (LPS)-stimulated RAW macrophages *in vitro* [24]. Here we investigate the potential of MSE to relieve UC symptoms in DSS-induced acute and chronic UC models in mice.

## Materials and methods

### Chemicals and reagents

DSS (molecular weight: 36–50 kDa) was purchased from MP Biomedicals, Inc. (Solon, OH), and 5-ASA (known as mesalamine) was from TCA America (Portland, OR). Roswell Park Memorial Institute (RPMI) 1640 medium, heat-inactivated fetal bovine serum (HI-FBS), and antibiotics (100 U/ml penicillin + 100 μg/ml streptomycin) were purchased from Thermo Fisher Scientific, Inc. (Waltham, MA). All other chemicals were purchased from Sigma-Aldrich Co. (St. Louis, MO) unless otherwise noted.

### Formulation of MSE

MSE was prepared following the protocol developed in our laboratory [24]. Briefly, moringa seeds (obtained from Jamaica Moringa Farmers’ Association, Kingston, Jamaica) were ground to a fine powder using a blender (VitaMix 5200; Cleveland, OH) and mixed with ambient temperature Millipore water in a ratio of seeds (1 g) to water (3 ml). The mixture was then placed on a shaker and incubated for 2 h at 37°C. After incubation, ethanol was added to the mixture in a ratio of seeds (1 g) to ethanol (12 ml), stirred, and filtered. Subsequently, solvents were removed from the extract by rotary evaporation and lyophilization before being stored at -20°C. MSE powder used in this study contained 47% (*w/w*) of MIC-1. Additional constituents of MSE consisted of moisture (13.92%), protein (crude; 18.27%), fat (crude; 1.76%), fiber (crude; 0.60%), ash (4.01%) and carbohydrate (62.4%, including 45.4% total sugar) (NJFL, Inc; Trenton, NJ).

### Animals and experimental protocols

All animal protocols used in this study were approved by the Institutional Animal Care and Use Committee at Rutgers, The State University of New Jersey (No. 14–014), and followed federal and state laws. Male C57BL/6J mice at 7 wks of age (average weight: 20–22 g) were purchased from Jackson Laboratories (Bar Harbor, ME) and acclimatized to the animal facility for 1 wk with *ad libitum* access to drinking water and a rodent chow diet (Purina 5001; LabDiet, St. Louis, MO) prior to experiments. Mice were initially housed 4 animals per cage, and maintained under standardized conditions (22 ± 2°C, 12-h light/dark cycle, and 50–60% humidity). Experiments began with mice at 8 wks of age, randomly assigned to the experimental groups, because this age is optimal for successful and reproducible UC induction by DSS [25]. For this study, two types of DSS-induced UC mouse models with features of acute and chronic UC conditions were utilized. UC induction protocols and experimental designs for each UC model are described below and summarized in Table 1.

**Table 1:** Experimental designs and protocols^a^.

	Acute UC model	Chronic UC model
**UC induction by DSS**	3.0% *(w/v)* DSS (5 d)	4 cycles of 2.5% *(w/v)* DSS for 3 d, with alternate autoclaved water for 6 d in between (30 d in total)
**Dosing method**	Oral gavage	Oral gavage
**Treatment period**	7 d	14 d
**Experimental** **design**	Group 1) Water/vehicle (healthy control) Group 2) DSS/vehicle (disease control) Group 3) DSS/MSE (MSE treatment; 150 mg/kg bw of MSE) Group 4) DSS/5-ASA (positive control; 50 mg/kg bw of 5-ASA)	

^a^ Modified based on Dey et al., 2010 [26]

### DSS-induced acute UC model

After acclimation, 32 mice were randomly divided into 4 experimental groups as follows based on their bw (n = 8/group): healthy control (water/vehicle), disease control (DSS/vehicle), MSE treatment (DSS/MSE), or positive control (DSS/5-ASA). Acute UC was induced in mice (except healthy control) by replacing their drinking water with autoclaved water containing freshly prepared 3.0% (*w/v*) DSS (*ad libitum*) for 5 d, which, along with a chronic UC induction method described below, was previously confirmed in our laboratory as the highest tolerable DSS concentration/duration capable of developing an acute or chronic UC model without mortality in 7 wk old, male C57BL/6J mice [26]. Mice in healthy control were littermates that received autoclaved water instead, and housed and processed identically during UC induction. Following UC induction, treatments were administered daily to all mice via oral gavage in a volume of 5 ml/kg bw with animal feeding needles (20G x 1.2”; Cadence, Inc., Staunton, VA) for 7 consecutive d. Treatments included vehicle (autoclaved water), 150 mg/kg bw of MSE, or 50 mg/kg bw of 5-ASA. The level of 150 mg/kg bw MSE (containing ~70 mg/kg bw MIC-1) was chosen based on our previous study with phenethyl isothiocyanate (PEITC; ~70–75 mg/kg bw), a structurally and functionally-related isothiocyanate derived from winter cress, showing an efficacy at this level in attenuating bowel inflammation in DSS-induced UC mice [26]. This MSE dose was also confirmed to be safe from our recent toxicity study in rats. Mice were allowed free access to autoclaved drinking water and chow pellets during the time.

### DSS-induced chronic UC model

After acclimation, 24 mice were assigned to 4 weight-balanced experimental groups (n = 6/group) as described above. Chronic UC was induced in mice by 4 cycles of 2.5% (*w/v*) DSS in drinking water for 3 d, which was alternated with 6 d of autoclaved water (*ad libitum*) in between, resulting in a total of 30 d for the entire induction period. Subsequently, treatments (vehicle, MSE, or 5-ASA) were orally delivered to mice daily for 14 consecutive d, and mice had free access to autoclaved water and chow during the time.

Throughout the entire experimental periods with both UC models, bw and food consumption were recorded daily or every 2 d. Intakes of DSS-containing water were also monitored daily to confirm that mice received sufficient amounts of DSS to induce UC. At the end of each experiment, mice were anesthetized with CO_2_ and euthanized by decapitation.

### Disease activity index (DAI) assessment

DAI was scored following the criteria described in Table 2 in order to validate the progression of UC in both models. The DAI score was calculated as a sum of the scores for % bw change, intestinal bleeding, stool consistency, and rectal prolapse. % bw change was calculated as: [(bw each d–initial bw) / initial bw] x 100. To score intestinal bleeding, bloody stool was confirmed by either visible observation or the fecal occult bleeding test (FOBT) using a commercially available kit (Sure-Vue™; Thermo Fisher Scientific, Inc., Waltham, MA).

**Table 2:** Disease activity index (DAI) for clinical evaluation of DSS-induced UC in mice^a^.

Score	% bw change	Intestinal bleeding (FOBT)^b^	Stool consistency	Rectal prolapse
**0**	Gain, loss < 1%	No color change	Normal	None
**1**	Loss 1 to <5%	-	-	-
**2**	Loss 5 to <10%	Color change	Loose	Observed prolapse
**3**	Loss 10 to <15%	-	-	-
**4**	Loss ≥ 15%	Gross bleeding	Diarrhea	Extensive prolapse

^a^ Modified from Dey et al., 2010 [26]

^b^ Fecal occult bleeding test

### Tissue isolation, measurements, and preparations

At the time of sacrifice in acute and chronic UC experiments, the spleen and colon were dissected from each mouse and measured within 5–10 min postmortem. Individual spleen tissues were weighed (mg). The colons, connected to the cecum, were isolated by separating them from the ileocecal junction of the small intestine and the distal rectum of the anus, and thoroughly flushed with cold PBS (pH 7.4; supplemented with 1% antibiotics) using a ball tip dosing needle attached to a 10 ml syringe to remove feces and blood. After pushing out remaining colon contents with a blunt forceps, the colons were straightened and measured in length (cm). The colons were then separated from cecum and weighed. Subsequent to these measurements, the colons were opened by longitudinal incision, and washed three times in cold PBS (pH 7.4; supplemented with 1% antibiotics) for a complete removal of fecal matter, from which only middle-to-distal colon segments were used for further analyses. These colon segments were further cut into 3 pieces longitudinally, each of which was individually processed for organ culture, quantification of gene expression, and histopathological assessments.

### *Ex vivo* colon organ culture

Approximately 1/3 of washed middle-to-distal colon segments were kept in cryogenic tubes filled with cold RPMI 1640 medium (supplemented with 1% antibiotics) on ice until delivery to the cell culture facility. On culturing, each colon specimen was transferred into a separate well of a 48-well microtiter plate containing 1 ml/well of RPMI 1640 medium (supplemented with 10% FBS and 1% antibiotics), and incubated at 37°C with 5% CO_2_ for 18 h. Supernatants were collected, centrifuged at 2,500 rpm for 10 min at 4°C, and stored at -80°C until analyses of colonic pro-inflammatory biomarkers.

### Histopathological evaluation of UC

Another 1/3 of middle-to-distal colon segments were subjected to histopathological assessment. The colonic sections were fixed in 4% paraformaldehyde overnight at 4°C, which were then washed with PBS (pH 7.4), dehydrated through ethanol gradients, and paraffin embedded. The embedded blocks were sectioned (5 μm), and the samples were then stained with hematoxylin and eosin (H&E). Subsequently, all colonic sections were scored in an investigator-blinded manner relating to the severity of colonic damage and inflammation using light microscopy with a Retiga 1300 CCD camera (Q-imaging; Burnaby, BC, Canada) and an Eclipse E800 microscope (Nikon Instruments Inc., Lewisville, TX) with QC-Capture imaging software under X20 and X400 magnification and air for 20x/0.75 N.A. The severity of UC was evaluated using the histopathological scoring system with total score range from 0 to 11 (Table 3), according to the matrices previously defined by Laroui et al. (2012) [27].

**Table 3:** Histopathological scoring system^a^.

Score	Severity of inflammation	Crypt damage	Ulceration
**0**	Rare inflammatory cells in the lamina propria	Intact crypt	0 foci of ulceration
**1**	Increased numbers of granulocytes in the lamina propria	Loss of basal 1/3 of crypt	1–2 foci of ulceration
**2**	Confluent inflammatory cells extended to submucosa	Loss of basal 2/3 of crypt	3–4 foci of ulceration
**3**	Transmural extension of the inflammatory infiltration	Loss of entire crypt	Confluent or extensive ulceration
**4**	-	Change in epithelial surface caused by erosion	-
**5**	-	Confluent erosion	-

^a^ Laroui et al., 2012 [27]

### Quantification of colonic pro-inflammatory biomarkers

From the supernatant of *ex vivo* colon cultures, pro-inflammatory chemokine, keratinocyte-derived chemokine (KC), and pro-inflammatory cytokine, TNF-α, were quantified using the mouse CXCL1/KC DuoSet ELISA kit and the mouse TNF-α Quantikine ELISA kit, respectively. Myeloperoxidase (MPO), a marker for neutrophil infiltration in colonic epithelial tissues, was also measured using the mouse MPO DuoSet ELISA kit. All ELISA kits were purchased from R&D Systems, Inc. (Minneapolis, MN), and utilized according to manufacturer’s protocols. NO production was analyzed in duplicates using the Griess Reagent System (Promega Corp., Madison, WI) following the manufacturer’s protocol. Nitrite levels (μM) in individual samples were determined at 520 nm by comparison with a standard curve of nitrite (0.1 M NaNO_2_; 0 to 100 μM). Throughout these analyses, absorbance was read using a Synergy HT™ microplate reader (BioTek Instruments, Inc., Winooski, VT), and individual concentrations obtained from each colon sample were normalized by the measured colon tissue weight (g).

### Fecal collection and quantification of fecal lipocalin-2 (Lcn-2)

At the end of treatment periods of acute and chronic UC experiments, fecal samples were collected by taking individual mice out of their cages and collecting 7 fecal pellets per mouse in sterile cryogenic vials, which were snap-frozen on dry ice immediately, and stored at −80°C until further processing. Quantification of fecal Lcn-2 was performed as described by Chassaing et al. (2012) [28] with slight modifications. Briefly, 100 mg of frozen fecal samples were reconstituted in 1 ml of PBS (pH. 7.4) supplemented with 0.1% (*v/v*) Tween 20, which were homogenized for 3 min using a 1600 MiniG® homogenizer (SPEX SamplePrep, Inc., Metuchen, NJ) to obtain a homogenous fecal suspension. The suspension was centrifuged at 12,000 rpm at 4°C for 10 min and the clear supernatant was used to quantify Lcn-2 levels using the mouse Lcn-2 DuoSet ELISA kit (R&D Systems, Minneapolis, MN) following manufacturer’s instructions. The level of fecal Lcn-2 was expressed as Lcn-2 (ng)/feces (g).

### Total RNA isolation, purification, and cDNA synthesis

Individual colon tissue samples (~50 mg) were homogenized for 3 min with 2.8 mm stainless steel beads and Qiazol reagent (Qiagen Inc., Valencia, CA) using a 1600 MiniG® homogenizer (SPEX SamplePrep, Inc., Metuchen, NJ). From the colon homogenates, total RNA was isolated and purified using an RNeasy® Plus Universal mini kit (Qiagen Inc., Valencia, CA) according to manufacturer’s protocols. The purity and concentration of total RNA was measured spectrophotometrically at 260 nm using the NanoDrop® ND-1000 (NanoDrop Technologoes, Inc., Wilmington, DE), and only samples with sufficient purity (A_260/280_ ≥ 2.0) were subjected to the next step. cDNAs were synthesized from 5 μg RNA (per 25 μl reaction) using a High-Capacity cDNA Reverse Transcription kit (Applied Biosystems, Foster City, CA) with RNase inhibitor (Applied Biosystems, Foster City, CA), according to the manufacturer’s protocol. The thermal cycler program was set on a Mastercycler® epgradient (Eppendorf North America, Hauppauge, NY) as follows: 10 min at 25°C, 60 min at 37°C, 60 min at 37°C, 5 sec at 85°C, and final hold at 4°C.

### Quantitative real-time PCR (qPCR)

The synthesized cDNAs were diluted 50-fold or 100-fold, appropriately, and 2.0 μl of the dilution was used for qPCR with 5.0 μl of TaqMan® Fast Universal PCR Master Mix (2x, No AmpErase® UNG; Applied Biosystems, Foster City, CA), 0.5 μl of each primer, and 2.5 μl of nuclease-free H_2_O (Thermo Fisher Scientific, Inc., Waltham, MA) to a final reaction volume of 10.0 μl. Quantitative PCR amplifications were performed on a QuantStudio 3® Real-Time PCR System (Applied Biosystems, Foster City, CA) with the following thermal cycler profile: one cycle for 20 sec at 95°C, followed by 40 cycles of two-step procedure consisting of denaturation for 1 sec at 95°C and annealing extension for 20 sec at 60°C. Inventoried TaqMan primer sets (Thermo Fisher Scientific, Inc., Waltham, MA) were used for analyses of IL-1β (Mm00434228_m1), IL-6 (Mm00446190_m1), TNF-α (Mm00443258_m1), iNOS (Mm00440502_m1), zonula occludens-1 (ZO-1; Mm00493699_m1), claudin-1 (Mm00500912_m1), heme oxygenase 1 (HO1; Mm00516005_m1), NQO1 (Mm01253561_m1), and glutathione S transferase pi 1 (GSTP1; Mm04213618_gH), using either hydroxymethylbilane synthase (HMBS; Mm01143545_m1) or glyceraldehyde-3-phosphate dehydrogenase (GAPDH; Mm99999915_g1) as endogenous controls. All experimental samples were run in duplicates. For individual qPCR experiments, means of duplicate samples were taken, and relative quantification of target gene expression was normalized with respect to the average Ct value of the housekeeping gene, either HMBS or GAPDH, accordingly. Data were analyzed according to the comparative Ct method (2^*-*ΔΔCt^), where healthy control (water/vehicle) served as a calibrator and was assigned a value of 1.0. Consequently, all data are expressed as relative fold change between healthy control and other experimental groups.

### Statistical analysis

All statistical analyses were performed using SPSS statistical software package (version 11.5; SPSS Inc., Chicago, IL). Data were tested for normality of distribution (Shapiro-Wilk). Log transformations were applied to data that were not normally distributed. Statistical significance among different groups was evaluated by one-way analysis of variance (ANOVA), followed by a *post-hoc* test: Duncan’s test or Tukey’s test. Data are expressed as mean ± SEM. A p value of <0.05 was considered statistically significant.

## Results

### MSE alleviates UC symptoms

**Fig 1:**
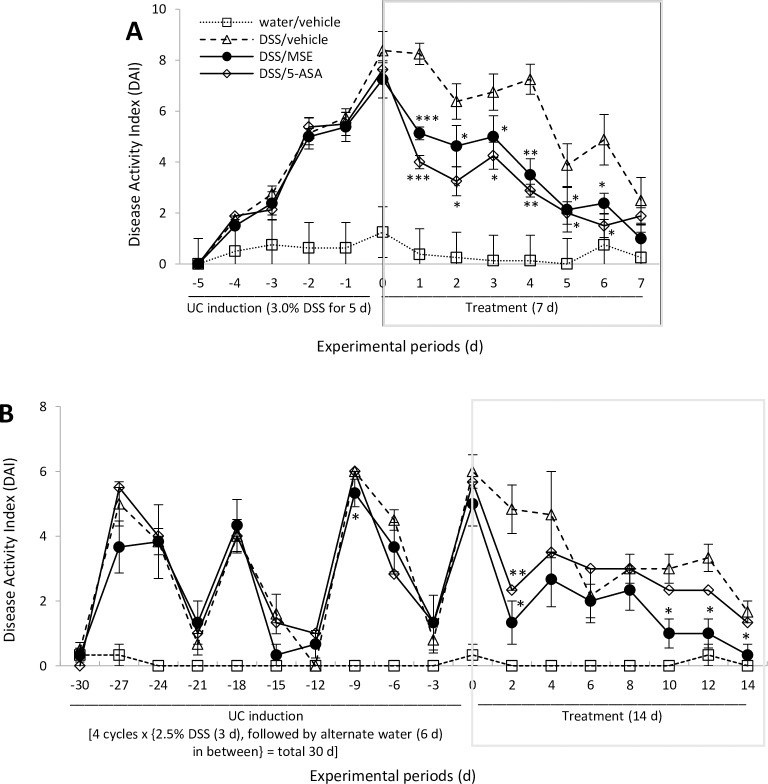
Effects of MSE on DAI scores in DSS-induced UC. **(A)** Acute UC (n = 8/group): data are shown at daily intervals throughout UC induction and treatment periods. **(B)** Chronic UC (n = 6/group): data are shown at 3-d intervals during UC induction and 2-d intervals during the treatment period. Experimental designs and disease induction procedures are defined in Table 1 and DAI scoring criteria in Table 2. Data are expressed as mean ± SEM. *p<0.05, **p<0.01, and ***p<0.001 compared to disease control (DSS/vehicle). 5-aminosalicylic acid (5-ASA); disease activity index (DAI); dextran sulfate sodium (DSS); moringa seed extract (MSE); ulcerative colitis (UC).

No animals died during acute or chronic UC periods. DAI score was calculated by combining scores of the following parameters: % bw change, intestinal bleeding, stool consistency, and rectal prolapse (see Table 2). Rectal prolapse was not observed in mice tested in either UC phase. Generally, mice receiving DSS manifested similar clinical symptoms seen in human UC, such as severe bw loss, diarrhea, and intestinal bleeding. By the end of UC induction, gradual increases of DAI scores were observed in acute (~7.75 times increase at d 5 induction) and chronic (~5.55 times increase at d 30 induction) UC models compared to healthy control (Fig 1A and 1B).

In chronic UC, particularly, a fluctuation of DAI scores was shown following the alternation of DSS and water, reflecting the relapse-remission pattern of UC symptoms (Fig 1B). In acute UC, both MSE and 5-ASA significantly decreased DAI scores compared to disease control for 6 consecutive d with a decrease starting from d 1 (-29.2% by MSE; -47.6% by 5-ASA) (Fig 1A). At d 7, however, no significant differences were found in DAI scores among any experimental groups, possibly due to a partial natural recovery from DSS-induced UC (Fig 1A). In chronic UC, MSE showed a strong trend towards reducing clinical symptoms compared to disease control starting from d 2 (-73.3%). The remission persisted throughout the treatment. At the end of treatment (d 14), the DAI score dropped almost to that of the healthy control (-93%) (Fig 1B). While 5-ASA and MSE showed similar trends in relieving UC symptoms, the 5-ASA effect was only significant at d 2 (Fig 1B), while the MSE effect was significant at d 2, 10, 12 and 14 (Fig 1B).

### MSE restores colon integrity in DSS-induced UC models

**Fig 2:**
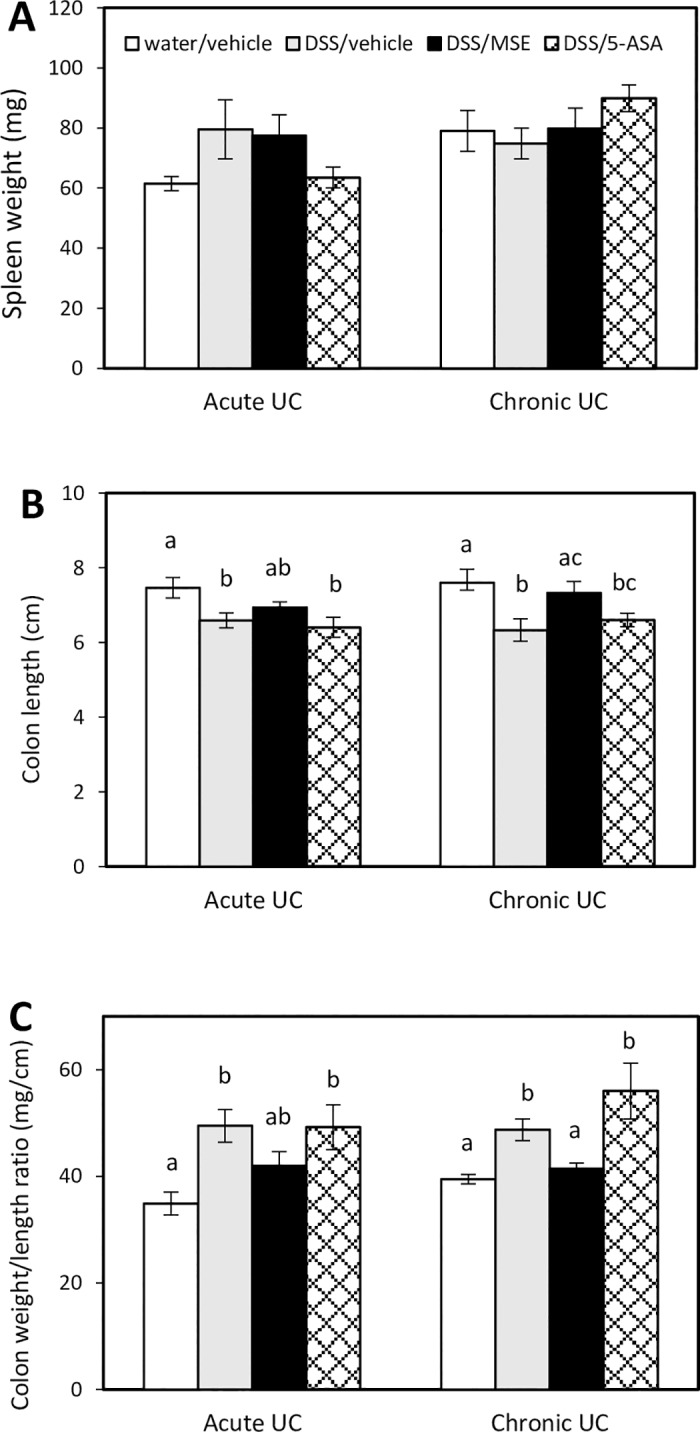
Effects of MSE on spleen and colon in DSS-induced UC. **(A)** Spleen weight (mg), **(B)** colon length (cm), and **(C)** colon weight/length ratio (mg/cm) were measured at necropsy of acute UC (n = 8/group) and chronic UC (n = 6/group) experiments. Experimental designs and disease induction procedures are defined in Table 1. Data are expressed as mean ± SEM. Different letters (a, b, c) indicate significant differences between groups at p<0.05. 5-aminosalicylic acid (5-ASA); dextran sulfate sodium (DSS); moringa seed extract (MSE); ulcerative colitis (UC).

Bw loss, rectal bleeding, and inflammation exerted during UC are associated with colon shortening and spleen enlargement [25, 29]. In this study, spleen weights were not affected by either DSS or treatments (MSE or 5-ASA) in both acute and chronic UC mice (Fig 2A).

However, mice in disease control had significantly shorter colons compared to healthy control (6.6 ± 0.2 cm vs. 7.5 ± 0.2 cm for acute UC; 6.3 ± 0.4 cm vs. 7.6 ± 0.4 cm for chronic UC). MSE showed a positive trend in reversing colon shortening (6.9 ± 0.1 cm for acute UC, not significant at p<0.05; and 7.3 ± 0.3 cm for chronic UC). 5-ASA did not recover colon shortening in either model (6.4 ± 0.3 cm for acute UC; 6.6 ± 0.2 cm for chronic UC) (Fig 2B). The colon weight/length ratio, an indicator of UC-associated colonic edema and inflammation [30], was significantly higher in disease control (49.5 ± 3.1 for acute UC; 48.7 ± 2.1 for chronic UC) and following 5-ASA treatment (48.7 ± 2.1 for acute UC; 55.9 ± 5.2 for chronic UC) compared to healthy control (34.9 ± 2.1 for acute UC; 39.5 ± 0.9 for chronic UC). MSE treatment reduced the ratio compared to disease control in both UC models (41.9 ± 2.7 for acute UC, not significant at p<0.05; and 41.5 ± 1.0 for chronic UC) (Fig 2C).

### MSE moderately lessens histopathologies associated with DSS-induced UC

**Fig 3:**
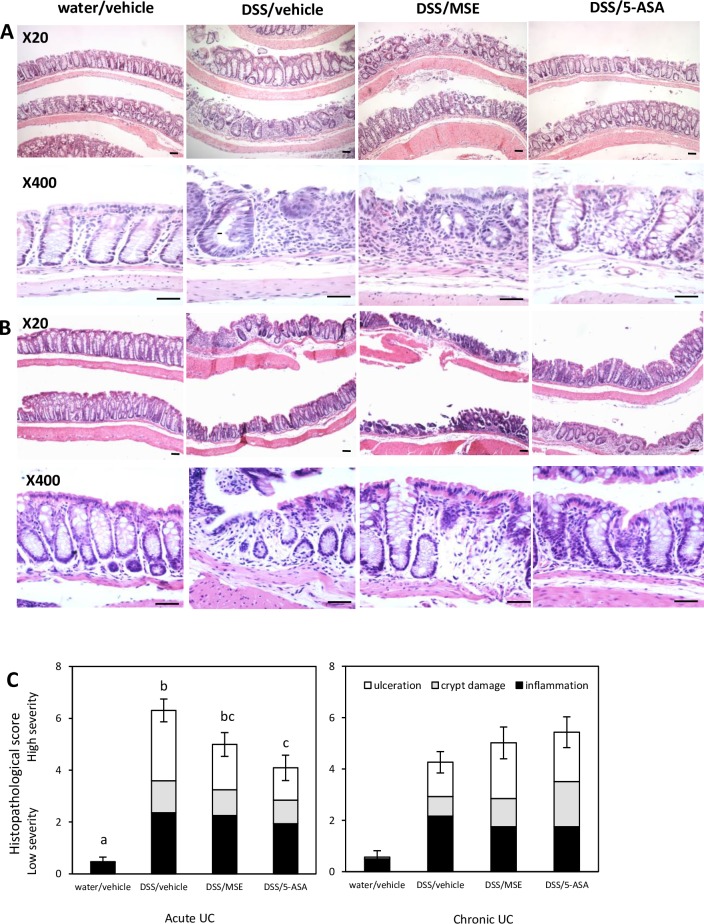
Effects of MSE on colon histopathology in DSS-induced UC. Representative histological observations of H&E-stained colonic sections of **(A)** acute UC mice (n = 8/group), and **(B)** chronic UC mice (n = 6/group). Experimental designs and disease induction procedures are defined in Table 1. **(C)** Associated colonic histopathological scores calculated as sum of scores of each parameter consisting of histopathological scoring system as described in Table 3. Histological images were acquired by X20 and X400 magnification, respectively (Scale bar = 25 μm). Data are expressed as mean ± SEM. Different letters (a, b, c) indicate significant differences between groups at p<0.05. 5-aminosalicylic acid (5-ASA); dextran sulfate sodium (DSS); hematoxylin and eosin (H&E); moringa seed extract (MSE); ulcerative colitis (UC).

The histopathological analysis was performed in H&E-stained distal colon sections of mice with DSS-induced acute and chronic UC. Microscopic images (X20 and X400 magnification) of representative colonic sections were taken for each group, and histopathological scores were assessed (Table 3). In both UC models, the healthy control groups showed intact epithelium with undamaged crypts and no ulcerative foci (Fig 3A and 3B).

In disease control mice, colonic sections exhibited severe crypt loss, extensive mucosal damage, and extended inflammatory cell infiltration into mucosal and submucosal compartments, as is commonly reported in the DSS-induced UC model. Extensive erosion and submucosal edema were also presented in the distal colon as the indication of an inflammatory phenotype in this group (Fig 3A and 3B). On the contrary, treatment with either MSE or 5-ASA resulted in less severe inflammation (relatively less extensive epithelial erosion) and development of a few regenerating foci compared to disease control in acute UC (Fig 3A). In chronic UC, neither MSE nor 5-ASA treatments measurably rescued chronic inflammation, as extensive mucosal damage and confluent ulcerative erosion were similarly observed in treated and disease control groups (Fig 3B). In the assessment of histopathological scores, all mice in DSS-treated groups showed a marked increase of the scores, which was significantly reduced by 5-ASA. MSE showed a trend in reducing histopathological scores in acute UC, which was not significant at p<0.05 (Fig 3C).

### MSE decreases secretion of colonic pro-inflammatory biomarkers and fecal Lcn-2 levels

**Fig 4:**
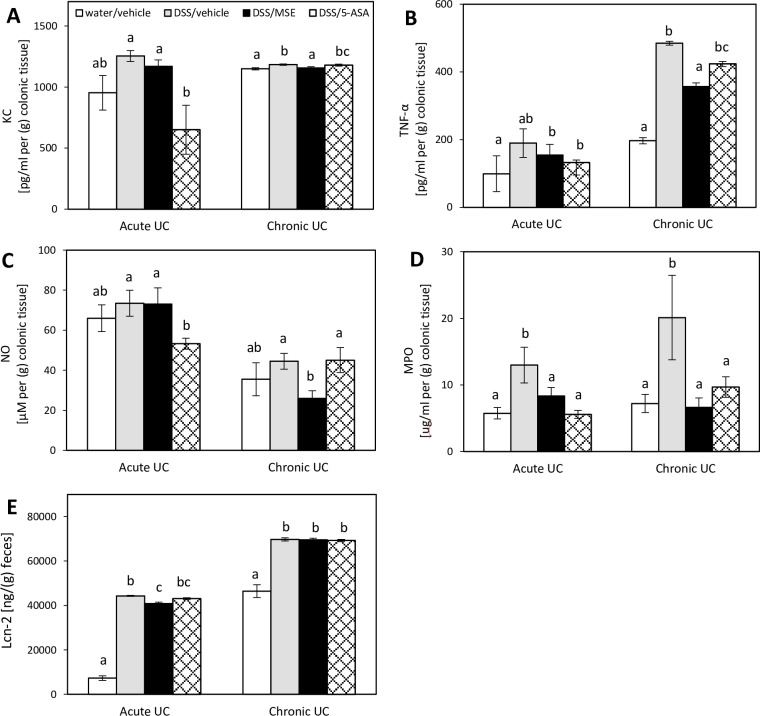
Effects of MSE on colonic pro-inflammatory biomarkers and fecal Lcn-2 levels in DSS-induced UC. Analyses of **(A)** KC, **(B)** TNF-α, **(C)** NO, and **(D)** MPO were performed in colon culture supernatants of acute UC (n = 8/group) and chronic UC (n = 6/group) mice. All concentrations were normalized to 1 g of the colon tissue weight. Fecal levels of **(E)** Lcn-2 were normalized to 1 g of the fecal weight. Experimental designs and disease induction procedures are defined in Table 1. Data are expressed as mean ± SEM. Different letters (a, b, c) indicate significant differences between groups at p<0.05. 5-aminosalicylic acid (5-ASA); dextran sulfate sodium (DSS); keratinocyte-derived cytokine (KC); lipocalin-2 (Lcn-2); myeloperoxidase (MPO); moringa seed extract (MSE); nitric oxide (NO); tumor necrosis factor-α (TNF-α); ulcerative colitis (UC).

Cytokines and chemokines are important mediators of the mucosal inflammation and immune responses during UC. In acute UC, compared to disease control, colonic production of KC, TNF-α, and NO were decreased by MSE (not significantly at p<0.05), whereas 5-ASA significantly decreased levels of KC (-48.1%) and NO (-27.4%) (Fig 4A–4C).

The levels of MPO were significantly lowered by both MSE (-35.7%) and 5-ASA (-57.0%) in the acute UC model (Fig 4D). In chronic UC, reductions in these markers were more obvious, showing that, compared to disease control, colonic productions of all tested pro-inflammatory markers were significantly lowered by MSE (-2.3% for KC; -26.4% for TNF-α; -41.8% for NO; -67.1% for MPO). The effect of 5-ASA was negligible in chronic UC, only showing a significant reduction in MPO (-51.9%) (Fig 4A–4D). Mice receiving DSS showed a dramatic increase of the fecal Lcn-2 level in both acute and chronic UC, which, in acute UC, was significantly reduced by MSE (-7.7%), but not by 5-ASA, compared to disease control (Fig 4E).

### MSE downregulates colonic expression of pro-inflammatory cytokines and iNOS

**Fig 5:**
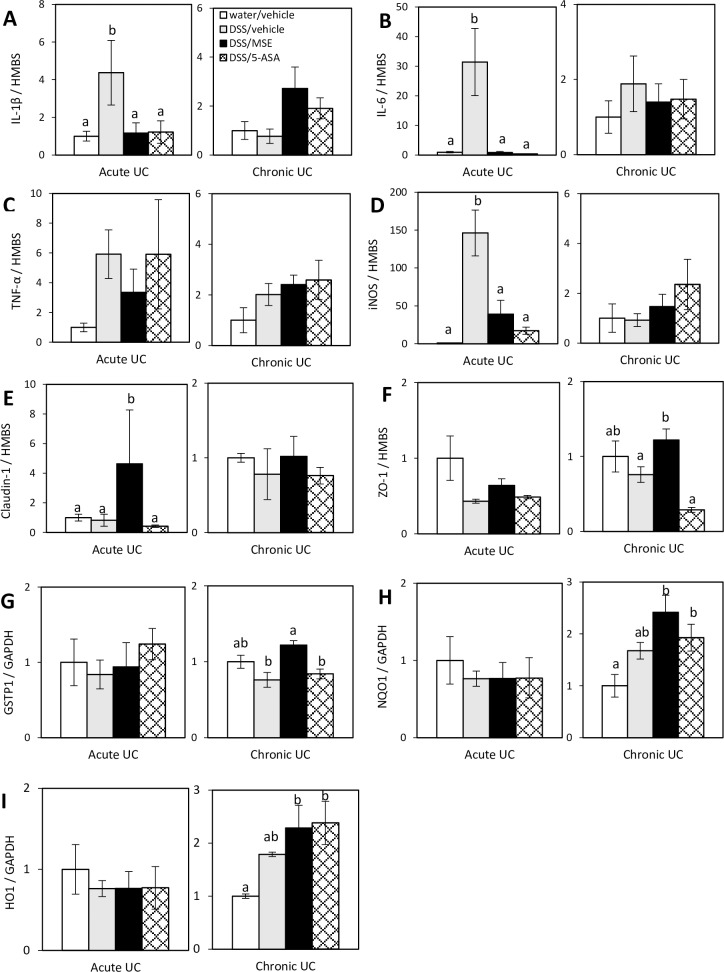
Effects of MSE on colonic expression of pro-inflammatory markers, tight-junction proteins, and Nrf2-mediated enzymes in DSS-induced UC. Gene expression of **(A)** IL-1β, **(B)** IL-6, **(C)** TNF-α, **(D)** iNOS, **(E)** claudin-1, **(F)** ZO-1, **(G)** GSTP1, **(H)** NQO1, and **(I)** HO1 was analyzed by qPCR in colon tissues of acute UC (n = 8/group) and chronic UC (n = 6/group) mice. All data are expressed as a relative fold change compared to healthy control (value = 1.0). Experimental designs and disease induction procedures are defined in Table 1. Data are expressed as mean ± SEM. Different letters (a, b) indicate significant differences between groups at p<0.05. 5-aminosalicylic acid (5-ASA); dextran sulfate sodium (DSS); glyceraldehyde-3-phosphate dehydrogenase (GAPDH); glutathione-S-transferase pi 1 (GSTP1); hydroxymethylbilane synthase (HMBS); heme oxygenase 1 (HO1); interleukin-1β (IL-1β); interleukin-6 (IL-6); inducible nitric oxide synthase (iNOS); moringa seed extract (MSE); NAD(P)H quinone oxidoreductase 1 (NQO1); tumor necrosis factor-α (TNF-α); ulcerative colitis (UC), zonula occludens-1 (ZO-1).

Pathological process of UC is closely related to the increased level of pro-inflammatory cytokines that can also activate iNOS. We examined the effect of MSE on colonic expression of pro-inflammatory cytokines including IL-1β, IL-6, and TNF-α, as well as iNOS. In acute UC, compared to healthy control, all these pro-inflammatory marker genes were upregulated by DSS (up to ~150 times for iNOS). However, in the acute model, the expression of these pro-inflammatory marker genes was, in most cases, markedly lowered by MSE (-73.2% for IL-1β; -97.1% for IL-6; -43.4% for TNF-α, not significant at p<0.05; -73.2% for iNOS) compared to disease control (Fig 5A–5D).

5-ASA, similar to MSE, downregulated the expression of the pro-inflammatory genes, except for TNF-α (Fig 5A–5D). In chronic UC, neither MSE nor 5-ASA showed significant effects on pro-inflammatory marker genes (Fig 5A–5D).

### MSE elevates colonic expression of tight-junction proteins and Nrf2-mediated phase II detoxifying enzymes

UC is associated with increased intestinal permeability and decreased tight-junction protein expression, such as claudin-1 and ZO-1, in the inflamed mucosa [30]. DSS normally suppresses claudin-1 gene expression; however, MSE increased claudin-1 expression in both UC models and the effect was highly significant in the acute model (558%) (Fig 5E). A similar result was observed for ZO-1, showing decreased gene expression by DSS, which was upregulated by MSE, but not by 5-ASA, in acute (148%; not significant at p<0.05) and chronic UC (148%) compared to disease control (Fig 5F). Gene expression of phase II detoxifying enzymes, including GSTP1, NQO1, and HO1, are directly regulated by the Nrf2 transcription factor. Nrf2 can also modulate NF-κB-dependent pro-inflammatory signals [19]. In chronic UC, MSE led to upregulated gene expression of GSTP1 (160%), NQO1 (144%), and HO1 (128%), compared to disease control; however, only GSTP1 upregulation was significant (Fig 5G–5I). In addition, the expression of NQO1 and HO1 genes was significantly upregulated by MSE compared to healthy control (Fig 5H and 5I). In acute UC, DSS showed a trend towards reducing the Nrf2-regulated gene expression that was not affected by MSE or 5-ASA (Fig 5G–5I).

## Discussion

This study aimed to investigate the efficacy and mechanisms of action of MIC-1-enriched MSE in alleviating UC symptoms. Data indicate that oral administration of MSE at 150 mg/kg bw mitigated UC symptoms and associated biomarkers in both DSS-induced acute and chronic UC mouse models by modulating inflammatory and antioxidant response systems.

Acute UC is characterized by rapid onset of severe clinical symptoms within a relatively short induction period, whereas chronic UC represents less severe and more sustained inflammation developed through a slower but long-lasting induction period with possible flare-up and remission symptomatic cycles. DSS disrupts the integrity of the mucosal barriers [20], leading to increased permeability of luminal toxic substances into the lamina propria and submucosal compartments, resulting in secondary inflammation in the intestine [25]. In our study, two DSS-induced UC models were successfully established, with more severe UC symptoms observed in the acute than chronic UC model, reflected by higher DAI scores (~7x vs. ~5x higher DAI score in acute vs. chronic UC, respectively) compared to DSS-untreated mice. Oral delivery of MSE (150 mg/kg bw for 1 or 2 wks) promptly reduced the severity of UC symptoms in both UC models, including improvements in bw loss, stool consistency, and intestinal bleeding. MSE improved the integrity of colon tissues by correcting colon shortening, likely due to loss in crypt structure, and colon thickness, which is due to edema formation [31]. MSE also reduced the severity of colonic inflammation developed during UC, diminished epithelial erosion and regenerated a few foci in colonic tissues in acute UC. However, MSE failed to attenuate these histopathological signs of UC in the chronic UC model. This is, nevertheless, consistent with a previous report that complete healing of bowel ulceration may not be concomitant with clinical remission of UC symptoms, and with recommendations from previous clinical studies to measure mucosal restitution after 8 wks of treatments [32]. 5-ASA, an anti-inflammatory drug for treatment of mild-to-moderate severity of UC in humans, was used as a positive control with a recommended dose of 50 mg/kg bw [32]. 5-ASA produced a comparable DAI score to that of MSE in acute UC, but was less efficacious than MSE in chronic UC. Moreover, the impact of 5-ASA on colonic histopathological signs of UC was only evident in acute UC, and could not improve aberrant characteristics of colon tissues, like colon shortening, caused by DSS. Our observations in chronic UC mice corroborate previous findings that 5-ASA, albeit widely used as a first-line therapy for UC, is largely ineffective in chronic UC [33].

Our findings are consistent with published reports on powerful anti-inflammatory effects of various isothiocyanates [12, 15, 16] and previous observations that orally administered PEITC (~75 mg/kg bw) or sulforaphane (25 mg/kg bw), isothiocyanates from *Brassica* vegetables that have the same pharmacophore (N = C = S) as MIC-1, could also relieve acute and chronic symptoms of DSS-induced UC in mice [14, 26]. Our companion paper published in this journal [24] demonstrates that MIC-1 is the main anti-inflammatory component of MSE. MIC-1 was more effective than curcumin (one of the most potent anti-inflammatory phytochemicals [34]) in reducing the production of NO and expression of iNOS, IL-1β, and IL-6 in LPS-stimulated RAW macrophages. Additionally, MIC-1 significantly upregulated the expression of Nrf2-mediated phase II detoxification genes (GSTP1, NQO1, and HO1) in these cells more effectively than curcumin. MSE was more potent than curcuminoid-enriched turmeric extract in reducing carrageenan-induced paw edema in rats [24]. Taken together with our current observations, the available data suggests that MIC-1 is the bioactive component of MSE responsible for its therapeutic effects in UC.

Imbalanced production of cytokines is a part of UC pathophysiology. These cytokines form a complex network that mediates mucosal/submucosal inflammation and influences the integrity of epithelium [35, 36]. An increase of pro-inflammatory cytokines, including IL-1β, IL-6, TNF-α, and interferon-γ (IFN-γ), is associated with disease activity and development of all UC [37–39]. Meanwhile, KC serves as a chemoattractant for neutrophils [40], whereas MPO is a direct indicator of neutrophil infiltration into the colon and serves as a surrogate marker of UC-related inflammation [23]. Administration of anti-KC antibodies reduced neutrophil trafficking into the colons of mice with DSS-induced UC [41], indicating a key role of KC in colonic recruitment of neutrophils. Lcn-2, also referred to as neutrophil gelatinase-associated lipocalin (NGAL) or siderocalin, is a bacteriostatic protein mainly released from neutrophils at sites of inflammation [42], and its level in serum, colon, and fecal samples is elevated in multiple models of UC and in human IBD [43, 44]. iNOS-generated NO is a signaling molecule in inflammation, infection, and cancer [45]. iNOS knockout mice exhibited a substantial reduction of DSS-induced colonic injury compared to wildtype [45]. A positive correlation between NO and increased pro-inflammatory cytokines (TNF-α, IL-6, IL-17, IL-12, and IFN-γ) is described in IBD patients [46]. We also observed increased production and/or gene expression of pro-inflammatory markers (IL-1, IL-6, TNF-α, iNOS, NO, KC, MPO, and Lcn-2) in both UC models. MSE treatment generally reduced these markers, although the effect of MSE differed in acute vs. chronic UC models and in protein vs. gene expression levels. The main reason of the latter observation (TNF-α and NO/iNOS) might be due to the different colon segments from each animal that were used for each analysis. Since the extent of colonic inflammation would not be identical among different segments of the colon in UC mice, some inconsistency could occur between protein and gene expression levels. Similarly, a previous study showed that PEITC downregulated colonic IL-1β gene expression in DSS-induced UC mice [26]. These results are consistent with the well-documented anti-inflammatory effects of various isothiocyanates, which are mediated, at least in part, by Nrf2 activation [46].

Tight-junction proteins, such as ZO-1, occludins, and claudins, form physiologically active barriers in intestinal epithelial cells that can be disrupted in IBD [30, 47, 48]. For example, the loss of ZO-1 protein was observed in DSS-induced UC mice [30]. Our data showed that, while DSS treatment led to some downregulation of claudin-1 and ZO-1 genes, MSE restored or upregulated these genes, consistent with its observed therapeutic effects in UC.

Oxidative stress is both a consequence of UC [49] and one of its contributing factors [50]. Our data suggest that MSE possibly protects against chronic colonic inflammation by lowering oxidative stress through upregulation of antioxidant/phase II detoxifying enzymes, in particular, GSTP1. GSTP1 is the most abundant isoform of glutathione S transferase (GST) in the colon [49], catalyzing the conjugation of xenobiotics to glutathione [51], and DSS-induced UC mice exhibited downregulation of GST gene expression [51]. We suggest that the upregulation of antioxidant enzymes observed in our study might be the result of MIC-1-mediated activation of Keap1/Nrf2/ARE (antioxidant response element) metabolic pathway [19, 52], similar to that caused by sulforaphane in DSS-induced UC [14]. Studies have shown that various isothiocyanates, such as sulforaphane, PEITC and benzyl isothiocyanate, induce ARE genes by activating the nuclear translocation of Nrf2 [52, 53], which subsequently reduces oxidative stress and inflammation [13, 19, 54]. Nrf2 knockout attenuated the anti-inflammatory effects of PEITC and curcumin in macrophages *ex vivo* [55]. Therefore, MIC-1 and other isothiocyanates are likely responsible for interactions with the Keap1/Nrf2 complex and further downstream processes [56]. Our study did not measure the direct effect of MIC-1 or MSE on Nrf2. However, changes in the expression of the downstream ARE genes regulated by Nrf2, such as GSTP1, support the role of Nrf2 in mediating the observed effects.

In conclusion, MSE, enriched with the MIC-1, its main bioactive, ameliorated pathological events associated with acute and chronic UC. Anti-inflammatory and antioxidant activities of MSE are likely associated with Nrf2-mediated anti-inflammatory/antioxidant signaling pathway. This study supports the therapeutic potential of MSE for prevention and treatment of UC and should be further explored in human studies.
